# Latitudinal Diversity Gradients (LDGs) and macroalgal microbiomes: A chimera of biotic and abiotic effects?

**DOI:** 10.1111/jpy.13208

**Published:** 2021-12-07

**Authors:** Linda A. Amaral‐Zettler

**Affiliations:** ^1^ NIOZ Royal Netherlands Institute for Sea Research P.O. Box 59 Den Burg 1790 AB The Netherlands; ^2^ Department of Freshwater and Marine Ecology Institute for Biodiversity and Ecosystem Dynamics The University of Amsterdam Amsterdam 1090 GE The Netherlands; ^3^ The Josephine Bay Paul Center for Comparative Molecular Biology and Evolution Marine Biological Laboratory Woods Hole Massachusetts 02543 USA

**Fig. 1:**
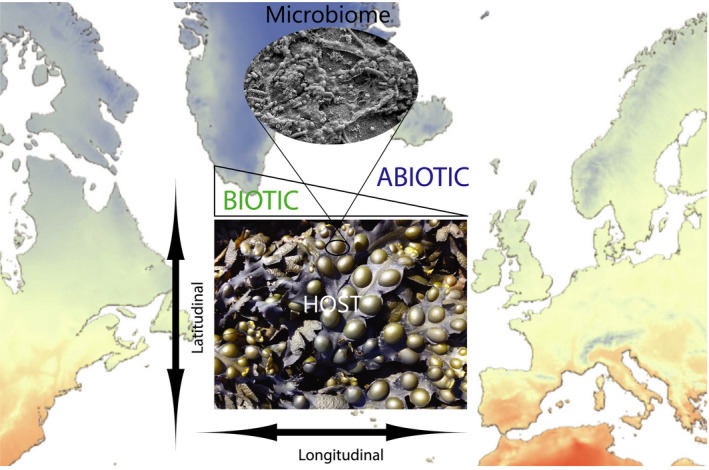
Brown macroalga *Fucus vesiculosus* and its microbiome provide a powerful platform for testing macroecological theories in biogeography. (Photograph/image credits: *Fucus* image—M.C. de Oliveira; *Fucus* microbiome SEM—L.A. Amaral‐Zettler and E.R. Zettler; ESRI basemap with Mean July Land Temperature layer added; Esri, Garmin, FAO, NOAA, WorldClim; Hijmans, R.J., S.E. Cameron, J.L. Parra, P.G. Jones and A. Jarvis, 2005. Very high resolution interpolated climate surfaces for global land areas. *Int*. *J. Climatol*. 25: 1965–78).

Latitudinal diversity gradients (LDGs) are among the best known and well‐studied biogeographic patterns in ecology (Wallace 1878, Hillebrand 2004, Brown 2014). The myriad of explanations for these patterns rivals the number of debates that has followed the development and testing of this theory. In a study of the microbiome of the brown macroalga *Fucus vesiculosus* (Fig. 1), Capistrant‐Fossa et al. (2021) explore the LDGs on either side of the Atlantic Ocean using amplicon sequencing of the V4 hypervariable regions of the rRNA gene followed by minimum entropy decomposition (MED) analysis (Eren et al. 2015). This unique model system is attractive because this macroalga's distribution—being a habitat‐ forming organism—stretches from the polar to the subtropical intertidal zones of both sides of the Atlantic Ocean acting to serve as a natural replicated experiment. The authors found latitudinal differentiation but no increase in species richness in the subtropics versus microbiomes in specimens sampled from sites in the north. This microbial‐centric study joins others that fail to find an LDG in microbiomes— questioning the ubiquity of this theory.

Previous work examining LDG and Rapoport's Rule in seaweeds (not their microbiomes) on European (Atlantic) and the temperate Pacific coasts also revealed mixed results: Atlantic populations displayed increasing species richness toward the tropics, whereas Pacific populations displayed the opposite trend (Santelices and Marquet 1998). These authors argued that their findings were consistent with Rapoport's Rescue Hypothesis, wherein small geographic ranges are linked to high diversity regions and low growth rates when compared to species with large geographic ranges as a general rule. Following definitions proposed by Ladau and Eloe‐Fadrosh (2019), the Capistrant‐Fossa study considered the spatial grain of 30 m transects along 16 sites (seven in the western Atlantic and nine in the eastern Atlantic), a temporal grain of two days in July, and a phylogenetic grain at the amplicon sequencing variant (ASV) level, while their spatial extent spanned 6,000 km, their temporal sampling extent was conducted over two years (summer of 2015 and 2016), and they targeted the domain Bacteria.

Whereas a justified aspect of this study was the choice of sample collection in the summer for all sites to account for effects of photoperiod, from a temporal perspective, given that alpha diversity in microbial communities peaks in the temperate areas in the winter (Ladau et al. 2013), an open question is whether a similar pattern exists in macroalgal microbiomes when temporal scales are expanded beyond a single season. From a phylogenetic grain perspective, the study focused on abundant ASVs to search for the “core” microbiome of *Fucus* across the latitudinal gradients. Studies on abiotic substrates (e.g., microplastics) have shown that rare taxa account for the “core” microbiome of plastic particles collected in different parts of the Atlantic Ocean, and the Baltic and Mediterranean Seas (Scales et al. 2021). Atlantic microplastic microbiomes have also revealed a pattern of increased richness in low latitudes (Amaral‐Zettler et al. 2015). These two former studies considered operational taxonomic units at the 97% clustering level— choice of phylogenetic grain that may explain the differences in the resulting observations.

Unlike the afore‐mentioned microplastics and other abiotic substrates that float, however, the Capistrant‐Fossa et al. study focused on microbiomes of a hard substrate‐attached biotic host: the model benthic macroalga, *Fucus* *vesiculosus*. The influence of bacteria on the morphogenesis of macroalgae is a significant aspect to consider in this respect and makes for a fascinating dimension lacking in studies that consider only abiotic substrates. To this end, a striking finding from this research was the discovery of tissue‐specific bacteria. For example, *Ilumatobacte*r_t6062 and Alphaproteobacteria_or_fa_ge_t3536 occurred in holdfasts over all sampling locations, and to a lesser extent, *Granulosicoccus*_t3260 occurred in all vegetative tips and receptacles but was absent in some of the sampled holdfasts. Although the search for a “core microbiome” using the ASV‐approach was not definitive, these tissue‐specific ASVs suggest a possible symbiotic role for bacteria in host morphology and physiology.

More pronounced in this study, however, were abiotic factors shaping the microbiome of *Fucus* *vesiculosus*. Whereas the genus *Granulosicoccus* was a quasi‐core member of the   microbiome of this seaweed, its presence correlated with cooler sea surface temperatures, based on environmental modeling done in the study. This finding has important implications for the geographic range of the host and the yet‐to‐be‐discovered role this quasi‐core microbiome member plays in host health, given future climate warming scenarios.

Lastly, the study leveraged unique historical range data for the host to determine that North‐South patterns in host ranges mirrored those of ASVs in the NW Atlantic. Parallel latitudinal microbial community structure and presence of common ASV's across the Atlantic sampling sites highlighted the non‐random nature of the study findings. Untangling host phylogeographic signal from biotic and abiotic influences remains an undertaking for future pursuit.
